# 

**Published:** 1839-10

**Authors:** 


### CONTENTS OF No. XVI.

OF THE

### British and Foreign Medical Review.

OCTOBER, 1839.

### PART FIRST.—Analytical and Critical Reviews.

PAGE

ART. I.—Leçons sur les Phénomènes Physiques de la Vie, professées au Collége de France. Par M. MAGENDIE. Recueillies par C. JAMES et G. FUNEL . 309
Lectures on the Physical Phenomena of Life, delivered at the College of France. By M. MAGENDIE, and collected by C. JAMES and G. FUNEL.

ART. II.—1. Lehrbuch der Arzneimittellehre. Von Dr. C. G. MITSCHERLICH . 353
A Manual of Materia Medica. By Dr. C. G. MITSCHERLICH.
2. The Elements of the Materia Medica; comprehending the Natural History, Preparation, Properties, &c. of Medicines. By JONATHAN PEREIRA, F.R.S. ib.
3. Die Neuesten Entdeckungen in der Materia Medica. Von Dr. J. H. DIERBACH ib.
The latest Discoveries in the Materia Medica. By Dr. J. H. DIERBACH.
4. Von dem Wirkungen der Gebräuchlichen Metalle auf den menschlichen Organismus überhaupt, und als Heilmittel; und dem Kupfersalmiak Liquor, und andern Kupferpräparaten als solcheninsbesondere. Von Dr. KÖCHLIN ib.
Of the Effects of the Metals in common use upon the Human Organism generally, and as Remedial Agents; and of the Cupro-Ammoniacal Liquor, and other preparations of Copper, as such, in particular. By Dr. J. R. KÖCHLIN.
5. Physiologisch-Therapeutische Untersuchungen überdas Veratrin. Von Dr. F. A. FORCKE . . . . . . ib.
Physiological and Therapeutical Researches concerning Veratrine. By Dr. FORCKE.

ART. III.—Die geburtshülfliche Auscultation. Von Dr. HERM. FRANZ NAEGELE 365
Obstetric Auscultation. By HERMAN FRANCIS NAEGELE, M.D.

ART. IV.—Das Hirn des Negers, mit dem des Europäers und Orang-Outangs verglichen. Von Dr. F. TIEDEMANN . . . . 374
The Brain of the Negro compared with that of the European and the Orang-Outang. By Dr. F. TIEDEMANN.

ART. V.—1. Beiträge zur operativen Orthopädik, oder Erfahrungen über die subcutane Durchschneidung verkürzter Muskeln und deren Sehnen. Von Dr. STROMEYER 384
Contributions to Operative Orthopædia; on the Subcutaneous Division of Contracted Muscles and their Tendons. By Dr. LOUIS STROMEYER.
2. Handbuch der Plastischen Chirurgie. Von Dr. EDUARD ZEIS . . ib.
Manual of Plastic Surgery. By Dr. EDWARD ZEIS, of Dresden.
3. Mémoire sur la Section du Tendon d'Achille dans le Traitement des Pieds-bots. Par M. BOUVIER . . . . . 385
Memoir on the Section of the Tendo-Achillis in the treatment of Club-foot. By M. BOUVIER.
4. A Treatise on the Nature of Club-foot, and analogous Distortions, including their Treatment. By W. J. LITTLE, M.D. . . . . ib.

ART. VI.—Recherches Medico-physiologiques sur l'Electricité Animale. Par J. F. COUDRET, M.D. . . . . . . 406

Medico-physiological Researches on Animal Electricity. By J. F. COUDRET, M.D.

ART. VII.—1. Handwörterbuch der gesammten Chirurgie und Augenheilkunde. Herausgegeben von ERNST BLASIUS . . . . 413
Dictionary of General and Ophthalmic Surgery. Edited by Dr. E. BLASIUS.
2. The Cyclopædia of Practical Surgery. Edited by WILLIAM B. COSTELLO, M.D. (Art. Aneurism, by JAMES WARDROP, M.D.) . . . ib.

ART. VIII.—1. Diagnostisch-praktische Abhandlungen aus dem Gebiete der Medicin und Chirurgie; durch Krankheitsfälle erläutert. Von Dr. LÖWENHARDT . 419
Diagnostic and practical Essays on Medical and Surgical Subjects. By Dr. LÖWENHARDT. Part II.
2. Versuche für die praktische Heilkunde. Von FERDINAND JAHN . . ib.
Essays on Practical Medicine. By FERDINAND JAHN. Part I.

§

PAGE

Art. IX.—1. Deux Mémoires, en réponse à cette question—"Faire connoitre les Analogies et les Differences qui existent entre le Typhus et la Fièvre Typhoide." Par MM. C. E. S. G. de Claubry et J. J. H. Montault . 428
On the Analogies and Differences between Typhus and Typhoid Fever. By MM. Gaultier de Claubry and Montault.
2. A Report founded on the cases of Typhoid Fever, or the common continued Fever of New England. By James Jackson, m.d. . . . ib.
3. A Short Treatise on Typhus Fever. By G. L. Roupell, m.d. . . ib.
4. Etudes Cliniques sur divers points de l'Histoire des Fièvres Bilieuses et Typhoides. Par le Docteur H. C. Lombard . . . . . ib.
Clinical Remarks on certain points in the History of Bilious and Typhoid Fevers. By Dr. Lombard.
5. Der Typhus und dessen Erscheinungen, oder die Typhoseptosen, pathogenetisch und therapeutisch erläutert. Von L. Buzorini, m.d. . . ib.
Typhus and its Phenomena, or the Typhoseptoses; pathologically and therapeutically illustrated. By L. Buzorini, m.d.
6. Das Blutfieber, vorzuglich in seiner Verbindung mit einigen Krankheiten des Darmkanals; pathogenetisch und therapeutisch erläutert. Von F. Kehrer. ib.
Blood-Fever, especially in its Connexion with certain Diseases of the Alimentary Canal; pathologically and therapeutically illustrated. By Dr. Kehrer.

Art. X.—A Treatise on the Diseases of the Heart and Great Vessels, and on the Affections which may be mistaken for them; comprising the Author's View of the Physiology of the Heart's Action and Sounds. By J. Hope, m.d. f.r.s. 448

Art. XI.—The Accoucheur; a Treatise on Protracted Natural Labours, Suspended Animation in new-born Infants, Uterine Hemorrhage, &c. By John Craig 467

Art. XII.—1. Lehrbuch der Physiologie des Menschen. Von Dr. F. Arnold . 476
System of Human Physiology. By Dr. Frederic Arnold.
2. Lehrbuch der pathologischen Physiologie. Von Dr. Wilhelm Arnold . ib.
System of Pathological Physiology. By Dr. W. Arnold.

Art. XIII.—Medical Notes and Reflections. By Henry Holland, m.d. f.r.s., &c. 482

Art. XIV.—On the Physiological Inferences to be deduced from the Structure of the Nervous System in the Invertebrated Classes. By W. B. Carpenter, m.d. 506

Art. XV.—1. Selections in Pathology and Surgery; or, an Exposition of the Natnre and Treatment of Local Disease, exhibiting new pathological views, and pointing out an important practical improvement. By John Davies . 512
2. Practical Remarks on the use of Iodine locally applied in various Surgical Diseases and External Injuries. By John Davies, &c. &c. . . ib.

Art. XVI.—An Enquiry into the Morbid Effects of Deficiency of Food, chiefly with reference to their occurrence among the Destitute Poor; also Practical Observations on the Treatment of such cases. By R. B. Holland, m.d. . 524

---

### Part Second.—Bibliographical Notices.

Art. I.—Practical Observations on Diseases of Women. By William Jones, m.r.c.s. 529

Art. II.—The Unity of Disease analytically and synthetically proved: with Facts and Cases subversive of the received Practice of Physic. By S. Dickson, m.d. 531

Art. III.—First Annual Report of the Registrar-General of Births, &c. in England 532

Art. IV.—Physiology, for the Use of Elementary Schools. By C. A. Lee, m.d. 533

Art. V.—Six Mois de Séjour en Angleterre. Par Sirus Pirondi, d.m. . 534

Six Months' Residence in England in 1836. By Sirus Pirondi, m.d.

Art. VI.—The Surgeon's Vade Mecum: a Hand-book of the Principles and Practice of Surgery. By Robert Druitt, m.r.c.s. . . . 536

Art. VII.—1. Essay on Cruelty to Animals. By James Macaulay, m.a. . ib.

2. The Animals' Friend; or, the Progress of Humanity. No. VII. . . ib.

Art. VIII.—On the Enlisting, Discharging and Pensioning of Soldiers, with the Official Documents. By Henry Marshall, f.r.s.e. . . 537

Art. IX.—School Botany; or an Explanation of the Characters and Differences of the principal Natural Classes and Orders of Plants belonging to the Flora of Europe. By John Lindley, ph.d. f.r.s. . . . . . 538

PAGE

Art. X.—The Surgical Anatomy of the Arteries, and Descriptive Anatomy of the Heart; together with the Physiology of the Circulation in Man and Inferior Animals. By Valentine Flood, a.m. m.d. t.c.d. &c. . . 538

Art. XI.—The American Medical Library. Edited by R. Dunglison, m.d. . 539

Art. XII.—An Experimental Enquiry concerning the presence of Alcohol in the Ventricles of the Brain, after Poisoning with that liquid; together with Experiments, illustrative of the Action of Alcohol. By John Percy, m.d. ib.

Art. XIII.—Illustrations of Cutaneous Diseases. By Robert Willis, m.d. . 541

Art. XIV.—1. A Series of Anatomical Sketches and Diagrams, with Descriptions and References. By Thomas Wormald and Andrew M. M'Whinnie . 542
2. The Surgical Anatomy of the Groin, the Femoral and Popliteal Regions. By Thomas Morton . . . . . . ib.

Art. XV.—Observations on the Disorder of the General Health of Females called Chlorosis, showing the true cause to be entirely independent of Peculiarities of Sex. By Samuel Fox . . . . . ib.

Art. XVI.—The Collected Works of Sir Humphry Davy, Bart., ll.d. f.r.s. Edited by his Brother, John Davy, m.d. f.r.s. Vol. I. . . 545

Art. XVII.—Retrospect of the Progress of Surgical Literature for the Year 1838-9. By Messrs. Newnham, W. Wickham, and Salter . . . 546

Art. XVIII.—Political Medicine; the substance of a Discourse on Medicine, considered in its relations to Government and Legislation. By H. Maunsell, m.d. 547

Art. XIX.—Medical Lexicon; a new Dictionary of Medical Science, containing a concise Account of the various Subjects and Terms; with a Vocabulary of Synonymes, &c. By Robley Dunglison, m.d. &c. . . 548

---

### Part Third.—Selections from the British and Foreign Journals.

### I. THE FOREIGN JOURNALS.

#### ANATOMY AND PHYSIOLOGY.

M. Simon on the Physiology of Nutrition . . . . . 549
Dr. Marchand on the Production of Urea in the Animal Body . . 550
Dr. Bulard's Observations on the Plague . . . . ib.

#### PATHOLOGY, PRACTICAL MEDICINE, AND THERAPEUTICS.

Dr. Otto's Case of Apoplexia Cutanea . . . . . . 552
M. Lenepveu's Case of Chronic Farcy terminating in Acute Glanders . . ib.
M. Gouzee on Cod-liver Oil and Ray-liver Oil in Scrofulous Affections . 554
Dr. Albers on Fatty Disease of the Liver (Pimelosis Hepatica) . . ib.
Dr. Schlesier on the Medical Use of Alum, in Diseases of the Heart . . 555
M. Bonetti on Arteritis . . . . . . 556
Dr. Toulmouche's Experiments on Sulphuret of Antimony . . ib.
Dr. Malin's Contribution to Psychical Anthropology . . . 557

#### SURGERY.

Dr. Guerin on the Treatment of Lateral Curvatures of the Spine . . 557

Statistics of Tracheotomy . . . . . . . 558

Dr. Defer on Gangrene resulting from the employment of a Starched Bandage . ib.

Dr. Roux on the Superiority of M. Reynaud's Operation for Circocele . . ib.

Dr. Remak on Torsion of the Arteries . . . . . 559

M. Bouvier on Congenital Dislocations of the Femur . . . ib.

Dr. Löwenhardt on Chronic Hydrocephalus cured by Compression of the Head . 560

M. Malgaigne on the Treatment of Inguinal Hernia by Trusses . . 561

#### MIDWIFERY.

Singular Case of a Woman delivered of Five Children . . . 564
Drs. Bunsen and Kyll on Morbid Accumulation of the Liquor Amnii . ib.
Prof. Kilian on the Galvanic Obstetric Forceps . . . . 566
Singular Case of Malformation . . . . . . ib.
Dr. Schwabe's Case of Mole Pregnancy with Spontaneous Amputation . 567
Dr. Carganico's Case of Spontaneous Evolution . . . . ib.
Case of Difficult Delivery of Twins which were united by the Breast . . 568

PAGE

MEDICAL JURISPRUDENCE AND TOXICOLOGY.

Dr. Lachese, Jun. on the Action of Arsenious Acid . . . 568
M. Devergie on some New Signs of Suspension having taken place during Life . 572
Dr. Malle's New Process for the Separation of Arsenic in Organic Mixtures . 573
On Hydrated Peroxyde of Iron as an Antidote to Arsenic . . . ib.

MEDICAL STATISTICS.

Dr. Hoffman on Longevity and Mortality in Prussia . . . 575

II. THE AMERICAN AND COLONIAL JOURNALS.

ANATOMY AND PHYSIOLOGY.

Dr. Fisher's Contributions Illustrative of the Functions of the Cerebellum . 576
Dr. Bolton on the Physiology of the Iris . . . . 580
Prof. Dunglison on the Pulsations of the Fœtal Heart and the Umbilical Cord . 581

PATHOLOGY, PRACTICAL MEDICINE, AND THERAPEUTICS.

Dr. Somervail on the Therapeutic effects of Tobacco applied externally . 582

Dr. Evans's Account of the Asylum near Frankford, Pennsylvania . . 583

SURGERY.

Dr. Hosack's Cases of Sensitive Tumours of the Female Urethra, with Remarks . 585
Dr. Barton's Views and Teatment of an important Injury of the Wrist . 588
Dr. Billingslea on the Use of Iodine in Herpes . . . 591

MIDWIFERY.

Prof. Dunglison on the Temperature of the Vagina and Os Uteri during Labour ib.

MEDICAL STATISTICS.

Classification of Fracture Cases treated at the Native Hospital, Calcutta . 592

Diseases of House-Patients treated at the Native Hospital, Calcutta . . ib.

III. THE BRITISH JOURNALS.

ANATOMY AND PHYSIOLOGY.

Dr. Hake on the Structure and Functions of the Spleen . . 593
Dr. Alderson on the Difference of Colour in different parts of the Bodies of Animals ib.
Mr. Paget on the Blood-vessels of Tendinous Tissues . . . 594
Dr. Reid on the Effects of Lesion of the Trunk of the Ganglionic System of Nerves ib.

PATHOLOGY, PRACTICAL MEDICINE, AND THERAPEUTICS.

Dr. Simpson on the Origin of Hernia and Malformation in the Fœtus . . 594
Dr. Henderson on the Result of Experience in Sea-Scurvy . . . 595
Dr. Cahill's Observations on the Treatment of Delirium Tremens without Opium ib.
Mr. Dewar's History of a Singular Convulsive Disease . . . ib.
On the Treatment of Strangulated Hernia on the plan of Dr. O'Beirne . 596
Mr. Phillips's Case of Section of the Hamstring Tendons . . . ib.
On the Treatment of Prolapsus Uteri by means of the Actual Cautery and Caustics.
1. Mr. Phillips on a new and successful Method of treating Prolapsus Uteri . ib.
2. Letter from Dr. Evory Kennedy on the use of Caustics in Prolapsus Uteri . ib.

MIDWIFERY.

Mr. Terry's Case of Restoration of Animation in a Still-born Child . . 596

Dr. Churchill's Researches in Operative Midwifery . . . 597

MATERIA MEDICA AND CHEMISTRY.

Mr. Nichols's Economic Formula for Hydriodate of Potash . . . 597

TOXICOLOGY.

Experiments with the Wourali Poison. With Dr. Clanny's Remarks . . 597

PART FOURTH.—Medical Intelligence.

Provincial Medical Association . . . . . . 603

British Association for the Advancement of Science . . . 616

Books received for Review. . . . . . . 617

